# Migraine in Real-World Clinical Practice: Patient Burden, Healthcare Utilization and Unmet Needs—A Cross-Sectional Analysis of the I-GRAINE Study

**DOI:** 10.3390/brainsci16101040

**Published:** 2026-09-29

**Authors:** Piero Barbanti, Jesuély Spieckert de Souza, Giulia Fiorentini, Francesca Pistoia, Cecilia Camarda, Alberto Doretti, Gabriella Egeo, Florindo d’Onofrio, Cinzia Aurilia, Marco Bartolini, Simone Braca, Antonio Carnevale, Rosario Grugno, Massimo Autunno, Maurizio Zucco, Marco Russo, Simone Quintana, Stefano Caproni, Alfonsina Di Summa, Paola Scatena, Massimo Filippi, Monica Laura Bandettini Di Poggio, Cinzia Finocchi, Alessandra Cherchi, Paolo Solla, Riccardo Altavilla, Barbara Petolicchio, Annalisa Gai, Mattia Sansone, Rossana Terlizzi, Francesco Bono, Gennaro Saporito, Stefano Messina, Elisabetta Iannaccone, Roberto De Simone, Valentina Favoni, Martina Guarinoni, Giovanna Viticchi, Domenico Cosenza, Ludovica Ferraù, Laura Di Clemente, Prabha Cristina Ranchicchio, Steno Rinalduzzi, Ilaria Cetta, Sara Cazzulo, Francesco Tazza, Tommaso Ercoli, Carla Zanferrari, Francesca Gragnani, Pietro Antonio Bruno, Renata Rao, Fabio Valguarnera, Maria Albanese, Licia Grazzi, Fabrizio Vernieri, Alfonso Coppola, Carlo Colosimo, Francesca Cortese, Fabrizio Di Stani, Frederico Friedrich, Vanise Grassi, Carlo Tomino, Stefano Bonassi, Annamaria Porreca, Paola Torelli, Sabina Cevoli, Italian Migraine Registry (I-GRAINE) Study Group

**Affiliations:** 1 Department of Human Sciences and Quality of Life Promotion, San Raffaele University, 00163 Rome, Italy; 2 San Raffaele, 67039 Sulmona, Italy; 3 School of Medicine, Pontifical Catholic University of Rio Grande do Sul (PUCRS), Porto Alegre 90619-900, Brazil; jesuely.souza@edu.pucrs.br (J.S.d.S.);; 4 Headache and Pain Unit, IRCCS San Raffaele Rome, 00163 Rome, Italygabriella.egeo@sanraffaele.it (G.E.); fdistani@gmail.com (F.D.S.); 5 Department of Biotechnological and Applied Clinical Sciences, University of L’Aquila, 67100 L’Aquila, Italy; francesca.pistoia@univaq.it (F.P.);; 6 Department of Biomedicine, Neurosciences, and Advanced Diagnostics (BIND), University of Palermo, 90127 Palermo, Italy; 7 Laboratory of Neurosciences, Department of Neurology-Stroke Unit, Istituto Auxologico Italiano, IRCCS, 20149 Milan, Italy; 8 Stroke Unit and Headache Center, San Giuseppe Moscati Hospital, 83100 Avellino, Italy; 9 Neurological Clinic, Marche Polytechnic University, 60126 Ancona, Italy; 10 Department of Neurological Sciences, Reproductive Sciences and Odontostomatology, University of Naples “Federico II”, 80131 Naples, Italy; 11 Headache Center, San Filippo Neri Hospital, 00135 Rome, Italy; antonio.carnevale@aslroma1.it (A.C.);; 12 IRCCS Centro Neurolesi Bonino-Pulejo, 98125 Messina, Italy; 13 Department of Clinical and Experimental Medicine, University of Messina, 98125 Messina, Italy; 14 Centro Cefalee, UOC Neurologia, Azienda Ospedaliero Universitaria San Camillo-Forlanini, 00152 Rome, Italy; 15 Headache Center, Unit of Neurology, Neuromotor and Rehabilitation Department, Azienda USL-IRCCS Di Reggio Emilia, 42122 Reggio Emilia, Italysimone.quintana@ausl.re.it (S.Q.); 16 Neurology and Stroke Unit—Headache Center, Neuroscience Department, “S. Maria” University Hospital, 05100 Terni, Italy; 17 Headache Clinic, Neurology, Central Hospital of Bolzano, 39100 Bolzano, Italy; 18 Neurology Unit, San Camillo de Lellis, General District Hospital, 02100 Rieti, Italy; 19 Department of Neurology, IRCCS San Raffaele Scientific Institute, Vita-Salute San Raffaele University, 20132 Milan, Italy; 20 IRCCS Ospedale Policlinico San Martino, 16132 Genoa, Italy; 21 Neurological Division—San Paolo Hospital—ATS Liguria, 17100 Savona, Italy; 22 Unit of Clinical Pharmacology, Headache Center, AOU Cagliari, 09124 Cagliari, Italy; 23 SC Clinica Neurologica—AOU Sassari, 07100 Sassari, Italy; psolla@uniss.it; 24 Stroke Unit, Neurology Division, Headache Center, San Carlo Borromeo Hospital, ASST Santi Paolo e Carlo, 20153 Milan, Italy; 25 Headache Center, Sandro Pertini Hospital, ASL Roma 2, 00161 Rome, Italy; 26 Neurology and Headache Center, Cardinal Massaia Hospital, 14100 Asti, Italy; 27 Headache Center, San Giuseppe Moscati Hospital, 81031 Aversa, Italy; 28 Department of Neurosciences, Neurology and Stroke Unit, Bufalini Hospital, AUSL Romagna, 47521 Cesena, Italy; 29 NeuroB Center for Headache and Botulinum Toxin Therapy Clinic Center San Vitaliano, 88100 Catanzaro, Italy; 30 Istituto delle Scienze Neurologiche di Bologna, 40139 Bologna, Italy; 31 Headache Center, Unit of Neurology, Department of Medicine and Surgery, University of Parma, 43126 Parma, Italy; 32 UOSD Stroke Unit, AOU G. Martino, Department of Clinical and Experimental Medicine, 98125 Messina, Italy; 33 Headache Center and Neurology Unit, IRCCS San Raffaele Scientific Institute, 20132 Milan, Italy; 34 Neuroimaging Research Unit, IRCCS San Raffaele Scientific Institute, 20132 Milan, Italy; 35 Headache Center, Neurology Unit, University of Sassari, 07100 Sassari, Italy; 36 Department of Medical and Surgical Sciences, Institute of Neurology, Magna Graecia University, 88100 Catanzaro, Italy; 37 Neurology Unit, Headache Center Department of Continuity of Care and Frailty, ASST Spedali Civili Hospital, 25123 Brescia, Italy; 38 Neurology Unit, Department of Clinical and Experimental Sciences, University of Brescia, 25123 Brescia, Italy; 39 Centro Cefalee ASL3 Liguria, 16154 Genova, Italy; 40 Unit of Neurology, Department of Systems Medicine, University of Rome Tor Vergata, 00133 Rome, Italy; 41 Headache Center, Department of Neuroalgology, IRCCS Fondazione Istituto Neurologico Carlo Besta, 20133 Milan, Italy; 42 Headache and Neurosonology Unit, Fondazione Policlinico Campus Bio-Medico and Department of Medicine and Surgery, Università Campus Bio-Medico di Roma, 00128 Rome, Italy; 43 Department of Neurology, Headache Center G. Salvini Hospital, 20024 Garbagnate Milanese, Italy; 44 LUM University, “F. Miulli” Regional Hospital, 70021 Acquaviva delle Fonti, Italy; 45 Department of Neurology, Hospital São Lucas da PUCRS, Porto Alegre 90619-900, Brazil; 46 Department of Internal Medicine, Universidade Federal de Ciências da Saúde de Porto Alegre, Porto Alegre 90050-170, Brazil; 47 Department of Neurology, Santa Casa de Porto Alegre, Porto Alegre 90020-090, Brazil; 48 Scientific Direction, San Raffaele, 67039 Sulmona, Italy; 49 Clinical and Molecular Epidemiology, IRCCS San Raffaele Roma, 00163 Rome, Italy; stefano.bonassi@sanraffaele.it (S.B.);; 50 Department for the Promotion of Human Sciences and Quality of Life, University San Raffaele, 00163 Rome, Italy; 51 Azienda Ospedaliero-Universitaria di Parma, 43126 Parma, Italy

## Abstract

**What are the main findings?**

- Migraine care in Italy is characterized by a mismatch between healthcare resource utilization and clinical needs, with limited primary-care involvement, repeated specialist consultations, and healthcare nomadism.
- Healthcare resource utilization is substantial, including extensive diagnostic testing—predominantly brain imaging—as well as emergency department visits, detoxification cycles, and repeated access to headache centers.

**What are the implications of the main findings?**

- The findings support a reorganization of migraine care pathways, with stronger primary-care involvement, clearer referral pathways, and improved continuity of care.
- Greater adherence to guideline-based diagnostic and management strategies may reduce potentially avoidable healthcare utilization and promote more efficient, patient-centered migraine care.

## 1. Introduction

Migraine is a highly prevalent and disabling neurological disorder [1], characterized by recurrent headache attacks accompanied by symptoms such as photophobia, phonophobia, nausea, and vomiting. In addition, many patients experience other disabling symptoms not included in the ICHD-3 criteria, such as osmophobia, allodynia, cranial parasympathetic symptoms, dopaminergic symptoms, and dizziness, which further contribute to the overall disease burden [2]. In a relevant proportion of patients, migraine may progress and worsen over time, evolving toward high-frequency episodic or chronic forms, particularly in the presence of delayed diagnosis, suboptimal management, or inadequate preventive strategies [3].

While pain represents the most prominent feature of migraine attacks, the burden of migraine extends far beyond, affecting patients’ daily functioning, quality of life (QoL), and psychosocial well-being, with significant consequences in terms of activity limitation, avoidance behaviors, cephalalgiophobia, reduced occupational performance, and impaired interpersonal relationships [2,4,5].

In recent years, migraine management has substantially evolved, with the availability of migraine-specific, effective, and well-tolerated acute and preventive treatments that can reduce disease burden and improve patients’ QoL [6]. Despite the availability of effective therapies, migraine care remains inconsistent, and many patients do not have timely access to appropriate diagnostic and therapeutic pathways [7]. Limited disease awareness, misdiagnosis, fragmentation of care, and the use of ineffective or inappropriate treatments frequently characterize the early phases of the disease, contributing to delays in optimal management [8]. Fragmented care pathways and delayed access to appropriate care often result in a prolonged and complex patient journey, marked by repeated consultations, diagnostic procedures, treatment changes, and suboptimal outcomes, leading to increased healthcare utilization [9,10,11]. Despite increasing recognition of migraine as a major public health burden, substantial gaps persist between evidence-based recommendations and real-world clinical practice across healthcare systems. In Italy, despite the presence of two national scientific societies dedicated to headache disorders, active patient associations, and approximately 160 officially recognized headache centers and outpatient clinics, a similar gap remains evident.

In this context, implementation science plays a crucial role in bridging this gap by generating and translating real-world evidence into effective, sustainable, and patient-centered care pathways [12].

To address these unmet needs and to better characterize real-world care pathways, the Italian Migraine Registry (I-GRAINE) was established as a nationwide, multicenter initiative designed to systematically collect prospective real-world data from individuals with episodic (EM) and chronic migraine (CM) [2]. Within this framework, the present study aims to characterize prior healthcare utilization and HCRU among patients attending participating Italian headache centers, thereby providing real-world evidence relevant to clinical practice and health policy.

## 2. Materials and Methods

### 2.1. Study Design

This study is a cross-sectional analysis of enrollment data derived from the Italian Migraine Registry (I-GRAINE), a nationwide, multicenter prospective registry [2]. The registry was preregistered on ClinicalTrials.gov (ID: NCT07163416), started on 19 April 2021, and is currently ongoing. At registry initiation, all headache centers and outpatient clinics accredited by the Italian Neurological Association for Headache Research (ANIRCEF) and the Italian Society for the Study of Headaches (SISC) were invited to participate. Participation was voluntary: In total, 54 centers accepted the invitation, and 43 completed study initiation and contributed patients to the registry. All participating centers are affiliated with the Italian National Health System (Sistema Sanitario Nazionale, SSN) and span 13 Italian regions and 30 provinces. The present findings therefore refer to patients attending the participating Italian headache centers. Adult patients (≥18 years) diagnosed with EM or CM according to the International Classification of Headache Disorders, 3rd edition (ICHD-3) criteria were enrolled at the participating specialized headache centers [2]. On each outpatient clinic day, registry participation was proposed to the first eligible patient attending for a first visit and to the first eligible patient attending for a first follow-up visit. Recruitment continued until approximately 10% of the annual migraine caseload of each center was reached; therefore, no fixed numerical sampling interval was used. The numbers of potentially eligible patients who were not approached or declined participation were not systematically recorded, and a formal comparison with non-participating centers was not possible. Data were collected through a web-based platform (REDCap; Vanderbilt University, Nashville, TN, USA) [13] and included sociodemographic, clinical, and lifestyle variables, as previously described [2]. Healthcare-utilization variables were collected using predefined recall periods established by the I-GRAINE Registry. GP consultations and Emergency Room visits referred to the previous year; migraine-related diagnostic investigations referred to the previous three years; specialist consultations referred to consultations for migraine before entry to the headache center (lifetime); and detoxification cycles referred to the patient’s lifetime.

HCRU in acute and inpatient settings was assessed based on the frequency of Emergency Room (ER) visits for migraine management. For each resource category, the prescribing provider (GP or specialist) or patient-initiated access was identified, along with the funding source, distinguishing between services covered by the SSN and those paid out-of-pocket.

Prescription-source counts were recorded separately as specialist-prescribed, GP-prescribed, or patient-initiated examinations. In records with complete prescription-source information, these categories were mutually exclusive and summed to the total number of examinations for each modality. SSN-funded examination counts were analyzed separately using modality-specific complete-case denominators. Data collection relied on structured face-to-face interviews conducted by trained neurologists and was complemented by both patient-reported information and the review of available source documents, including headache center and specialists’ medical records as well as diagnostic test reports (e.g., neuroimaging, neurophysiological examinations), when accessible at the time of the visit [2].

### 2.2. Ethics Committee

All patients provided written informed consent prior to inclusion in the Registry. The study protocol was approved by the Ethics Committee of the coordinating center (IRCCS San Raffaele Roma; approval no. 16/int, 31 July 2020) and by the Ethics Committees of the participating centers. Full details on ethical approval procedures and data protection have been previously reported [2].

### 2.3. Statistical Analysis

Statistical analyses were performed using R software, version 4.5.1 (R Foundation for Statistical Computing, Vienna, Austria). The primary objective was to describe healthcare utilization patterns in the pooled I-GRAINE registry cohort. Comparisons between patients with EM and CM were secondary and exploratory and were intended to characterize observed differences within the study cohort rather than to estimate independent or causal effects of migraine subtype.

Continuous variables were summarized using median and interquartile range (IQR), whereas categorical variables were reported as absolute frequencies and percentages. Between-group comparisons were performed using Pearson’s chi-squared test or Fisher’s exact test, as appropriate, for categorical variables and the Wilcoxon rank-sum test (Mann–Whitney U test) for continuous variables. No multivariable adjustment for potential confounders or center-specific effects was performed. Accordingly, EM versus CM comparisons should be interpreted as unadjusted descriptive comparisons within the observed registry cohort.

Given the exploratory nature of these secondary comparisons and the absence of predefined confirmatory hypotheses, no formal adjustment for multiple testing was applied. Effect estimates and measures of uncertainty are reported where appropriate, and nominal *p*-values are interpreted cautiously as descriptive measures rather than confirmatory evidence of subtype-specific differences or equivalence. A nominal two-sided *p*-value < 0.05 was used for descriptive purposes.

Analyses were conducted using available data without imputation. Denominators therefore vary across variables according to the number of participants with non-missing information for each item. For key proportions, 95% confidence intervals were calculated using the Wilson score method.

For variables with substantial missing data, center-level patterns of missingness were evaluated among centers enrolling more than 10 patients. Missing-data proportions were calculated for each center and compared using Pearson’s chi-squared test of homogeneity. To assess whether overall missingness was disproportionately influenced by individual centers, a leave-one-center-out analysis was performed by sequentially excluding each center and recalculating the pooled proportion of missing observations.

## 3. Results

### 3.1. Sample Characteristics and Lifestyle

A total of 1630 patients were included in the analysis, of whom 81.7% (*n* = 1332) had EM and 18.3% (*n* = 298) had CM. The sample was predominantly female (85.4%). Median age was 47 years [IQR 36–55] overall, 47 [IQR 35–55] in EM, and 49 [IQR 40–57] in CM (nominal *p* = 0.005). Most participants were married or living under common law (58.8%), and 49.0% had more than 13 years of education. Median BMI was 22.7 kg/m^2^ [IQR 20.7–24.9] overall, with similar observed values in EM and CM (22.6 [20.6–24.9] vs. 23.0 [20.7–24.9], respectively; *p* = 0.284) (Table 1).

Lifestyle habits were broadly similar between groups with respect to smoking status, alcohol, and coffee intake. The proportion engaging in physical activity was 21.2% in CM and 26.8% in EM (*p* = 0.055). Medication overuse was more frequent in CM than EM (58.2% vs. 14.5%; *p* < 0.001), as were sleep disorders (39.1% vs. 30.6%; *p* = 0.006). More than one comorbidity was reported by 47.3% of patients with CM and 41.8% of those with EM (*p* = 0.086) (Table 1).

### 3.2. Healthcare Resource Utilization and Prior Healthcare Utilization Reported at Registry Enrollment

Overall, 33.4% (*n* = 504) of patients reported at least one GP visit for migraine, with similar observed proportions in EM and CM (Table 2). Overall, 64.0% of participants with available data reported ≥1 specialist consultation for migraine before entry to the headache center (lifetime), with comparable observed proportions in EM and CM (63.8% vs. 64.5%, respectively). Neurologists were the most frequently consulted specialists (80.4% of those reporting specialist consultations). Other consultations included ophthalmologists (26.3%), physiotherapists/osteopaths (19.1%), acupuncturists (13.8%), dentists/maxillofacial specialists (13.2%), otorhinolaryngologists (12.9%), physiatrists (11.4%), orthopedists (9.5%), dietitians/nutritionists (7.5%), internal medicine specialists (6.7%), and neurosurgeons (5.1%) (Table 2).

Repeated specialist consultations were reported before entry to the headache center. Among patients with visits recorded for each specialty, neurologists had the highest mean number of visits (3.22), with a median of 2 [IQR 1–3]. Specialty-specific median and IQR values are reported in Table 2. Among 1478 participants with valid data, the median age at first headache-center visit was 39 years [IQR 26–50] overall, 39 [IQR 26–49] in EM, and 39 [IQR 27–51] in CM (Wilcoxon rank-sum *p* = 0.402). Thirty implausible records were treated as missing for this derived variable. Overall, 52.2% of participants with available data reported no previous headache-center attendance. ER visits in the previous year and detoxification cycles over the lifetime were reported by 10.3% and 10.8% of participants, respectively.

### 3.3. Use of Diagnostic Investigations and Related Funding Reported at Registry Enrollment

The diagnostic-investigation item for the previous three years was populated for 1188 of 1630 participants (72.9%), of whom 765 reported at least one investigation (64.4%; 95% CI 61.6–67.1). The observed proportion was 64.4% in both EM and CM (Table 3). Brain imaging was reported by 96.9% of participants who underwent investigations (97.4% EM vs. 94.4% CM; *p* = 0.215). Brain magnetic resonance imaging (MRI) was the most frequent modality (83.8%), followed by brain computed tomography (CT; 22.6%) and brain magnetic resonance angiography (MRA; 21.7%). Missingness varied significantly across centers for both diagnostic investigations and specialist consultations (Pearson χ^2^, both *p* < 0.001); however, leave-one-center-out analyses indicated that the overall missingness pattern was not driven by any single center.

Spine imaging was reported in 16.2% of patients (12.0% spine MRI; 5.1% spine X-ray). Other diagnostic examinations were less frequent, including electroencephalography (9.0%), neurosonology studies (6.5%), and other neurophysiological examinations, such as evoked potentials (3.9%) (Table 3). Among records with available examination-count data, 1481 diagnostic examinations were reported across the eight recorded modalities. Examination counts were analyzed at the event level, whereas Table 3 reports the patient-level occurrence of each diagnostic modality. Examination-level prescription-source and SSN-funding results are presented in Table 4. Using modality-specific complete-case denominators, specialist prescription predominated for most examination types, accounting for 78.2% of brain MRI, 75.0% of brain CT, 89.9% of brain MRA, 90.6% of other neurophysiological examinations, 94.7% of neurosonology examinations, and 83.2% of EEG examinations. SSN funding ranged from 65.6% for spine MRI to 88.0% for other neurophysiological examinations. Prescription-source categories were mutually exclusive and summed to the total number of examinations in complete records; funding data used separate complete-case denominators (Table 4).

## 4. Discussion

In this large, multicenter, real-world analysis based on data from I-GRAINE, we provide a comprehensive characterization of prior healthcare utilization and HCRU among patients attending participating Italian headache centers. The I-GRAINE registry was specifically designed to capture real-world migraine care through standardized data collection in routine practice. Its implementation—based on predefined protocols, site initiation procedures, remote monitoring, continuous data validation, and monthly investigator meetings—supports a consistent and clinically detailed dataset [2].

**Figure 1:**
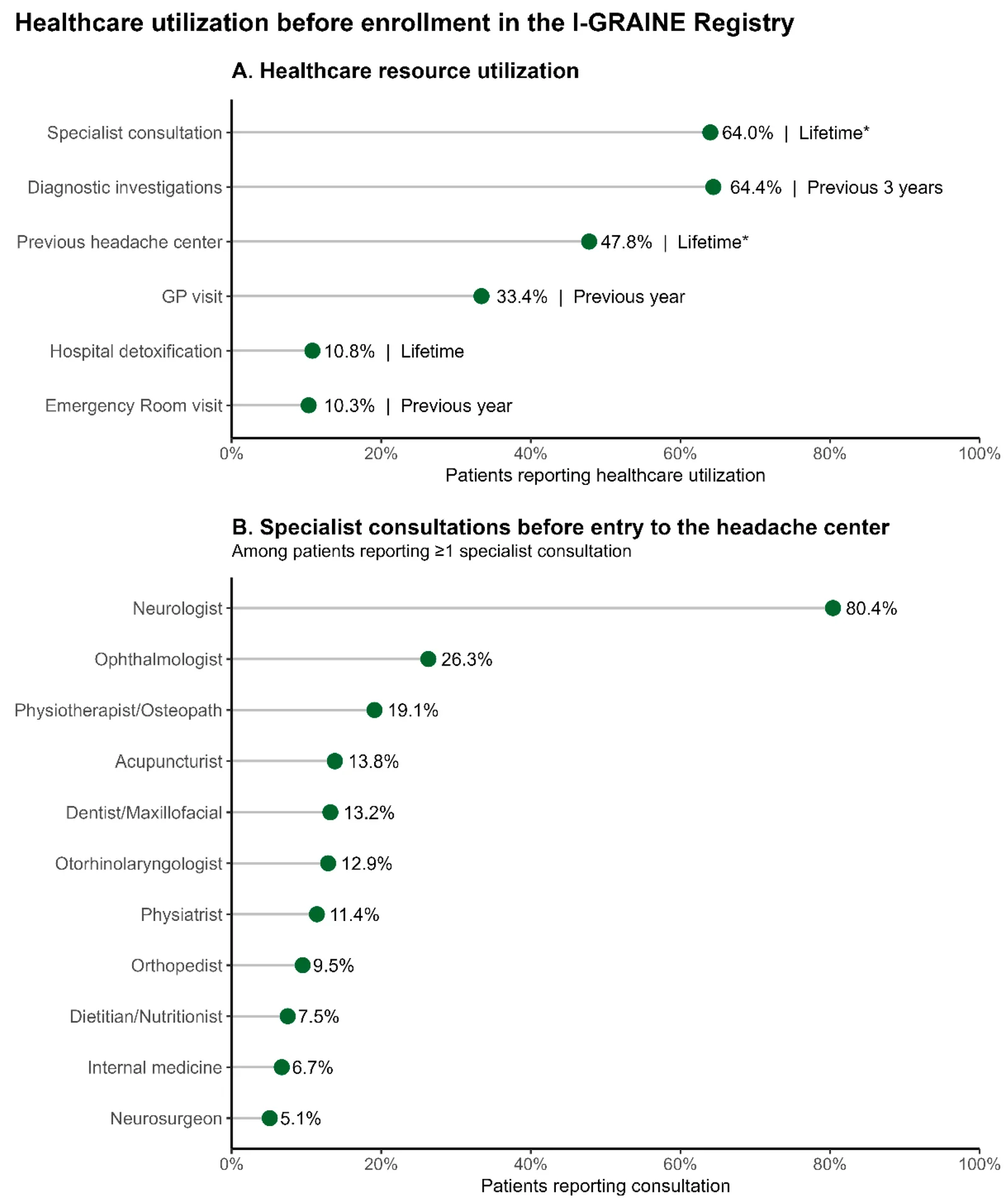
**Healthcare utilization reported before enrollment in the I-GRAINE Registry:** (**A**) Proportion of patients reporting selected healthcare resource utilization according to the predefined recall period for each resource. Specialist consultations refer to consultations for migraine before entry to the headache center (lifetime), whereas previous headache-center attendance refers to attendance before registry enrollment. (**B**) Types of specialists consulted among patients reporting at least one specialist consultation for migraine before entry to the headache center (lifetime). Recall periods differed across healthcare resource categories; therefore, proportions in Panel (**A**) should not be interpreted as directly comparable.

Overall, our findings delineate fragmented patterns of healthcare utilization, characterized by limited engagement with primary care, repeated specialist consultations, diagnostic testing, and multiple prior headache-center contacts (Figure 1). Because the available cross-sectional data do not provide a fully sequenced longitudinal diagnostic history, these repeated contacts should not be interpreted as evidence of a specific referral mechanism. These observations nevertheless highlight potential gaps between recommended care pathways and real-world practice.

Within this framework, several clinically relevant findings emerge. First, the role of primary care in migraine management appears to be limited. Only one-third of individuals with migraine reported at least one GP visit specifically for migraine management during the previous year, while nearly two-thirds reported at least one specialist consultation for migraine before entry to the headache center. Although the differing recall periods preclude direct comparison of GP and specialist utilization, the observed pattern is consistent with a limited role of primary care in migraine management and with previous Italian data indicating that a substantial proportion of patients with CM remain undiagnosed in primary care settings [14]. Potential contributors include under-recognition of migraine, variable confidence in its management within primary care, and suboptimal integration of primary care within structured headache pathways [14].

Second, participants reported repeated specialist consultations before entry to the headache center. Neurologists showed the highest number of reported visits per specialty, with a median of 2 [IQR 1–3]. Because these consultations were retrospectively reported at registry enrollment and were not linked to a complete sequence of diagnosis and referral dates, they cannot uniformly be interpreted as occurring before appropriate headache-center referral. Rather, they indicate repeated prior healthcare contacts within the registry recall window. Previous studies using explicitly sequenced diagnostic histories have also described complex and fragmented pathways to migraine diagnosis and specialist care [15,16,17].

Third, the median age at the first headache-center visit was 39 years [IQR 26–50] among participants with valid data. Approximately half of the participants had previously attended another headache center. These findings indicate multiple prior headache-center contacts in a substantial proportion of the cohort, but the available data do not establish the reasons for changing or consulting multiple centers, nor do they permit calculation of an individual-level interval from migraine onset to first headache-center access. Factors such as referral patterns, geography, waiting times, health-seeking behavior, and migraine-related stigma may influence access to specialist care, but these mechanisms were not directly measured in the present analysis [18,19,20,21].

Fourth, the diagnostic pathway appears largely oriented toward the exclusion of secondary causes. Neuroimaging was performed in most patients, often alongside CT, EEG, and other procedures of limited clinical value. This contrasts with current recommendations, which indicate that migraine diagnosis is primarily clinical and does not require imaging in the absence of red flags [22,23,24]. These findings likely reflect diagnostic uncertainty and contribute to delays in appropriate management. In addition, both patient-related factors, such as the need for diagnostic reassurance, and physician-related factors, including defensive medicine, may contribute to this approach.

Fifth, a proportion of patients accessed emergency care, with approximately 10% reporting at least one ER visit in the previous year. Although this may indicate suboptimal disease control, interpretation requires caution, as ER use may reflect not only disease severity but also factors such as attack-related anxiety, including cephalalgiophobia, or inadequate access to effective acute treatments [25]. The observed HCRU measures, including ER visits, detoxification cycles, and diagnostic procedures, should be interpreted as descriptive, unadjusted findings reported at registry enrollment rather than as temporally sequenced stages of care.

This study has limitations. As a multicenter registry-based cross-sectional analysis, it is subject to missing data, retrospective patient reporting, recall bias, and inter-center variability in clinical practice. In particular, the diagnostic-investigation field for the previous three years was blank for 442/1630 participants (27.1%); because this item was governed by REDCap branching logic, these blanks may include structurally non-applicable records as well as missing information, and available-case estimates should therefore be interpreted cautiously. Missingness varied across participating centers for diagnostic investigations and specialist consultations; however, leave-one-center-out analyses indicated that the overall level of missing data was not driven by any single center. Thus, missingness appears to reflect a broader limitation of registry data collection rather than being attributable to an isolated center, although center-related and patient-level selection mechanisms cannot be entirely excluded. The different recall periods used across healthcare-resource categories limit direct comparisons, and the available variables do not permit reconstruction of a complete longitudinal diagnostic journey or attribution of the reasons for repeated contacts with different headache centers. In addition, EM versus CM comparisons were unadjusted for potential confounders and did not explicitly model within-center correlation or between-center heterogeneity; consequently, inferential comparisons should be interpreted cautiously and should not be considered evidence of independent subtype effects or equivalence. Current EM versus CM status may also not represent migraine subtype during healthcare utilization accumulated historically over lifetime or multiyear recall periods. These limitations restrict generalizability beyond patients attending the participating Italian headache centers.

Despite these limitations, the I-GRAINE registry provides a structured and clinically detailed assessment of healthcare utilization in routine migraine care. By capturing comprehensive clinical and healthcare-utilization information through standardized registry procedures, it offers a useful framework for monitoring healthcare delivery and identifying gaps and unmet needs within specialized headache-care settings.

## 5. Conclusions

The I-GRAINE registry highlights persistent structural gaps in migraine care among patients attending participating Italian headache centers, characterized by a misalignment between HCRU and clinical needs. Addressing these gaps requires strengthening primary care, improving referral pathways, and promoting adherence to guideline-based diagnostic practices to reduce low-value resource use and improve patient outcomes. These findings provide real-world evidence relevant to the development of more efficient, equitable, and patient-centered models of migraine care within specialized headache-care settings. They also underscore the need for integrated, multidisciplinary care pathways and targeted educational interventions to optimize resource allocation and enhance the quality of migraine management.

## Figures and Tables

**Table 1:** Sociodemographic characteristics, lifestyle habits, and clinical comorbidities of patients with episodic and chronic migraine in the I-GRAINE Registry.

	All ^1^	Episodic Migraine ^1^	Chronic Migraine ^1^	*p* Value ^2^
Patients	1630	1332 (81.7%)	298 (18.3%)	
Female	1391/1629 (85.4%)	1135/1331 (85.3%)	256/298 (85.9%)	0.851
Age	47 [36–55]	47 [35–55]	49 [40–57]	0.005
Marital status				
Married/living under common law	956/1626 (58.8%)	759/1327 (57.2%)	197/298 (66.1%)	0.483
Single	533/1626 (32.8%)	453/1327 (34.1%)	80/298 (26.8%)	
Separated/divorced	88/1626 (5.4%)	71/1327 (5.3%)	17/298 (5.7%)	
Widower	15/1626 (0.9%)	13/1327 (1.0%)	2/298 (0.7%)	
Education (years)				0.389
<8	184/1577 (11.7%)	143/1284 (11.1%)	41/293 (14.0%)	
9–13	621/1577 (39.4%)	509/1284 (39.6%)	112/293 (38.2%)	
>13	772/1577 (49.0%)	632/1284 (49.2%)	140/293 (47.8%)	
Employment status				0.341
Employed	1130/1594 (70.9%)	910/1301 (69.9%)	220/293 (75.1%)	
Retired	76/1594 (4.8%)	67/1301 (5.1%)	9/293 (3.1%)	
Unemployed	120/1594 (7.5%)	99/1301 (7.6%)	21/293 (7.2%)	
Housework	143/1594 (9.0%)	118/1301 (9.1%)	25/293 (8.5%)	
Student	125/1594 (7.8%)	107/1301 (8.2%)	18/293 (6.1%)	
BMI	22.7 [20.7–24.9]	22.6 [20.6–24.9]	23.0 [20.7–24.9]	0.284
Menopause	430/1118 (38.5%)	332/890 (37.3%)	98/227 (43.2%)	0.119
Menopause age	50 [47.8–52]	50 [47–52]	50 [48–52]	0.707
Current smokers	258/1618 (15.9%)	201/1322 (15.2%)	57/295 (19.3 %)	0.068
Previous smokers	108/1618 (6.7%)	95/1322 (7.2%)	13/295 (4.4%)	0.797
Alcohol intake	95/1617 (5.9%)	82/1317 (6.2%)	13/298 (4%)	0.177
Coffee intake	90/1606 (5.6%)	71/1310 (5.4%)	19/296 (6.4%)	0.500
Physical activity	415/1610 (25.8%)	352/1313 (26.8%)	63/297 (21.2%)	0.055
Medication overuse	356/1578 (22.6%)	187/1290 (14.5%)	169/290 (58.2%)	<0.001
Sleep disorders	519/1612 (32.2%)	403/1316 (30.6%)	116/297 (39.1%)	0.006
≥1 comorbidity	697/1629 (42.8%)	556/1330 (41.8%)	141/298 (47.3%)	0.086

^1^ Denominators vary because of item-level missing data. Percentages were calculated using the available response denominator for each variable; no missing values were imputed. Categorical variables are expressed as absolute frequency (%), whereas continuous variables are expressed as median [IQR]; parentheses therefore indicate percentages, and square brackets indicate the interquartile range. ^2^ Pearson’s chi-squared test; Fisher’s exact test.

**Table 2:** Healthcare Resource Utilization (HCRU) and prior healthcare utilization reported at registry enrollment in patients attending participating Italian headache centers: data from the I-GRAINE Registry.

	All	Episodic Migraine	Chronic Migraine	*p* Value ^1^
Patients, n (%)	1630	1332 (81.7%)	298 (18.3%)	
≥1 GP visit for migraine during the previous year	504/1510 (33.4%; 31.0–35.8)	412/1230 (33.5%; 30.9–36.2)	92/280 (32.9%; 27.6–38.6)	0.838
≥1 specialist consultation for migraine before entry to the headache center (lifetime)	744/1163 (64.0%; 61.2–66.7)	604/946 (63.8%; 60.7–66.8)	140/217 (64.5%; 57.9–70.6)	0.853
Neurological	598 (80.4%)	489 (81.0%)	109 (77.9%)	0.763
Ophthalmological	196 (26.3%)	163 (27.0%)	33 (23.6%)	0.546
Physiotherapeutic/Osteopathic	142 (19.1%)	120 (19.9%)	22 (15.7%)	0.358
Acupuncture	103 (13.8%)	84 (13.9%)	19 (13.6%)	1.000
Dental/Maxillofacial	98 (13.2%)	77 (12.7%)	21 (15.0%)	0.419
Otorhinolaryngological	96 (12.9%)	81 (13.4%)	15 (10.7%)	0.495
Physiatric	85 (11.4%)	74 (12.3%)	11 (7.9%)	0.193
Orthopedic	71 (9.5%)	63 (10.4%)	8 (5.7%)	0.116
Dietetic/Nutritional	56 (7.5%)	45 (7.4%)	11 (7.9%)	0.860
Internal medicine	50 (6.7%)	41 (6.8%)	9 (6.4%)	1.000
Neurosurgical	38 (5.1%)	30 (5.0%)	8 (5.7%)	0.673
Number of specialist visits reported before entry to the headache center (lifetime), mean; median [IQR]				
*Neurologist*	3.22; 2 [1.00–3.00]	3.01; 2 [1.00–3.00]	3.98; 2 [1.00–5.00]	
*Internist*	1.61; 1 [1.00–1.75]	1.32; 1 [1.00–1.00]	2.15; 1 [1.00–2.00]	
*Dentist*/*Maxillofacial*	1.71; 1 [1.00–2.00]	1.59; 1 [1.00–2.00]	1.89; 1 [1.00–2.00]	
*Orthopedist*	1.31; 1 [1.00–1.00]	1.42; 1 [1.00–1.25]	1.12; 1 [1.00–1.00]	
*Ophthalmologist*	1.59; 1 [1.00–2.00]	1.50; 1 [1.00–2.00]	1.82; 1 [1.00–2.00]	
*Physiotherapist*/*Osteopath*	1.43; 1 [1.00–2.00]	1.41; 1 [1.00–2.00]	1.47; 1 [1.00–2.00]	
*Neurosurgeon*	1.63; 1 [1.00–2.00]	1.49; 1 [1.00–2.00]	1.94; 1 [1.00–2.00]	
*Otorhinolaryngologist*	1.73; 1 [1.00–2.00]	1.89; 1 [1.00–2.00]	1.49; 1 [1.00–2.00]	
*Dietitian*/*Nutritionist*	1.76; 1 [1.00–1.75]	1.51; 1 [1.00–1.00]	2.33; 1 [1.00–2.00]	
*Acupuncturist*	1.37; 1 [1.00–1.00]	1.39; 1 [1.00–1.00]	1.33; 1 [1.00–1.00]	
*Physiatrist*	1.36; 1 [1.00–1.00]	1.43; 1 [1.00–1.00]	1.18; 1 [1.00–1.00]	
Age at first headache-center visit, median [IQR]	39 [26–50]	39 [26–49]	39 [27–51]	0.402
Number of previously visited Headache Centers (lifetime)				
0	793/1518 (52.2%)	635/1234 (51.5%)	158/284 (55.7%)	
1	436/1518 (28.7%)	361/1234 (29.3%)	75/284 (26.4%)	
2	168/1518 (11.1%)	139/1234 (11.3%)	29/284 (10.2%)	
≥3	121/1518 (8.0%)	99/1234 (7.9%)	22/284 (7.7%)	
ER visits for migraine (last year)	163/1579 (10.3%; 8.9–11.9)	130/1291 (10.1%; 8.5–11.8)	33/288 (11.5%; 8.3–15.7)	0.498
Detox cycles (lifetime)	170/1569 (10.8%; 9.4–12.5)	137/1283 (10.7%; 9.1–12.5)	33/286 (11.5%; 8.3–15.8)	0.672

^1^ Pearson’s chi-squared test; Fisher’s exact test. For key HCRU proportions, values are reported as n/N (%; 95% CI). Specialist-type rows are reported as n (%). Specialist visit counts are reported as mean and median [IQR]. Age at first headache-center visit is reported as median [IQR] using valid observations. Variable-specific denominators reflect available data; no missing values were imputed. Square brackets indicate the interquartile range.

**Table 3:** Patient-level occurrence of migraine-related diagnostic investigations during the previous three years among patients attending participating Italian headache centers: data from the I-GRAINE Registry.

Diagnostic Investigations	All ^1^	Episodic Migraine ^1^	Chronic Migraine ^1^	*p*-Value ^2^
Patients with ≥1 instrumental examination within the last 3 years *	765/1188 (64.4%; 61.6–67.1)	622/966 (64.4%; 61.3–67.3)	143/222 (64.4%; 57.9–70.4)	0.994
Brain imaging	741/765 (96.9%; 95.4–97.9)	606/622 (97.4%; 95.9–98.4)	135/143 (94.4%; 89.3–97.1)	0.215
Brain MRI	641/765 (83.8%; 81.0–86.2)	526/622 (84.6%; 81.5–87.2)	115/143 (80.4%; 73.2–86.1)	0.240
Brain CT	173/765 (22.6%; 19.8–25.7)	143/622 (23.0%; 19.9–26.5)	30/143 (21.0%; 15.1–28.4)	0.719
Brain MRA	166/765 (21.7%; 18.9–24.8)	142/622 (22.8%; 19.7–26.3)	24/143 (16.8%; 11.5–23.8)	0.152
Spine imaging	124/765 (16.2%; 13.8–19.0)	99/622 (15.9%; 13.3–19.0)	25/143 (17.5%; 12.1–24.5)	0.617
Spine MRI	92/765 (12.0%; 9.9–14.5)	77/622 (12.4%; 10.0–15.2)	15/143 (10.5%; 6.5–16.6)	0.669
Spine X-ray	39/765 (5.1%; 3.8–6.9)	31/622 (5.0%; 3.5–7.0)	8/143 (5.6%; 2.9–10.7)	0.833
Electroencephalography	69/765 (9.0%; 7.2–11.3)	60/622 (9.6%; 7.6–12.2)	9/143 (6.3%; 3.3–11.5)	0.250
Neurosonology examinations	50/765 (6.5%; 5.0–8.5)	42/622 (6.8%; 5.0–9.0)	8/143 (5.6%; 2.9–10.7)	0.710
Other neurophysiological examinations	30/765 (3.9%; 2.8–5.5)	24/622 (3.9%; 2.6–5.7)	6/143 (4.2%; 1.9–8.9)	0.813

Values are reported as n/N (%; 95% CI); CIs were calculated using the Wilson score method. ^1^ Denominators vary according to available data. ^2^ Pearson’s chi-squared or Fisher’s exact test, as appropriate. * This row includes patients with available data; subsequent rows include only patients who underwent ≥1 examination.

**Table 4:** Examination-level analysis of prescription sources and Italian National Health Service (SSN) funding for migraine-related diagnostic investigations in the I-GRAINE Registry.

Examination-Level Prescription Source and SSN Funding				
Examination	Complete Prescription-Source Records, n	Total Examinations, n	Prescription Source: Specialist/GP/Patient-Initiated, n (%)	SSN-Funded, n/N (%)
**Brain MRI**	607	774	605 (78.2%)/106 (13.7%)/63 (8.1%)	577/738 (78.2%)
**Brain CT**	166	200	150 (75.0%)/34 (17.0%)/16 (8.0%)	162/197 (82.2%)
**Brain MRA**	157	178	160 (89.9%)/12 (6.7%)/6 (3.4%)	126/166 (75.9%)
**Spine X-ray**	38	43	20 (46.5%)/22 (51.2%)/1 (2.3%)	33/43 (76.7%)
**Spine MRI**	89	102	67 (65.7%)/22 (21.6%)/13 (12.7%)	63/96 (65.6%)
**Other neurophysiological examinations**	26	32	29 (90.6%)/1 (3.1%)/2 (6.3%)	22/25 (88.0%)
**Neurosonology examinations**	47	57	54 (94.7%)/2 (3.5%)/1 (1.8%)	44/57 (77.2%)
**EEG**	64	95	79 (83.2%)/12 (12.6%)/4 (4.2%)	81/93 (87.1%)

Prescription-source analyses were conducted at the examination level using complete-case data. Specialist-, GP-, and patient-initiated examinations were mutually exclusive and summed to the total number of examinations. Prescription-source values are reported as n (%); SSN-funded examinations are reported as n/N (%) using modality-specific complete-case denominators.

## Data Availability

The datasets generated and/or analyzed during the current study are not publicly available due to privacy and ethical considerations regarding the protection of participants’ data but are available from the corresponding author upon reasonable request, subject to appropriate approval.

## Abbreviations

BMI	body mass index
CM	chronic migraine
CT	computed tomography
EEG	electroencephalography
EM	episodic migraine
ER	emergency room
GP	general practitioner
HCRU	healthcare resource utilization
ICHD-3	International Classification of Headache Disorders, 3rd edition
MRA	magnetic resonance angiography
MRI	magnetic resonance imaging
NHS	National Health Service
QoL	quality of life
REDCap	Research Electronic Data Capture
SSN	Sistema Sanitario Nazionale

## References

[1] Collaborators GBDH (2025). Global, regional, and national burden of headache disorders, 1990–2023: A systematic analysis for the Global Burden of Disease Study 2023. Lancet Neurol..

[2] Barbanti P., Fiorentini G., Camarda C., Autunno M., Pistoia F., Aurilia C., d’Onofrio F., Egeo G., Carnevale A., Caproni S. (2025). Profiling the real-world migraine patient: Public health insights from sociodemographic, lifestyle, and clinical data in the Italian National Migraine Registry (I-GRAINE). J. Headache Pain.

[3] Buse D.C., Muenzel E.J., Zagar A.J., Mehrabadi A.S., Shapiro R.E., Kim G., Ashina S., Nicholson R.A., Lipton R.B. (2025). Rates and risk factors for migraine progression using multiple definitions of progression: Results of the longitudinal OVERCOME (US) study. Headache.

[4] Awaki E., Takeshima T., Matsumori Y., Hirata K., Miyazaki N., Takemura R., Osaga S., Tanizawa Y., Komori M. (2024). Impact of Migraine on Daily Life: Results of the Observational survey of the Epidemiology, Treatment, and Care of Migraine (OVERCOME [Japan]) Study. Neurol. Ther..

[5] Leonardi M., Raggi A. (2019). A narrative review on the burden of migraine: When the burden is the impact on people’s life. J. Headache Pain.

[6] Versijpt J., Paemeleire K., Reuter U., MaassenVanDenBrink A. (2025). Calcitonin gene-related peptide-targeted therapy in migraine: Current role and future perspectives. Lancet.

[7] Lanteri-Minet M., Leroux E., Katsarava Z., Lipton R.B., Sakai F., Matharu M., Fanning K., Adams A.M., Sommer K., Seminerio M. (2024). Characterizing barriers to care in migraine: Multicountry results from the Chronic Migraine Epidemiology and Outcomes–International (CaMEO-I) study. J. Headache Pain.

[8] Newman L.C., Lay C., Lipton R.B., Ailani J., Digre K.B., Caplan A., Singh N., Phillips H., Koh R., Warrick R. (2025). Navigating the patient journey in migraine prevention: An American Migraine Foundation position paper. Headache.

[9] Kim J.R., Park T.J., Agapova M., Blumenfeld A., Smith J.H., Shah D., Devine B. (2025). Healthcare resource use and costs associated with the misdiagnosis of migraine. Headache.

[10] Martelletti P., Schwedt T.J., Vo P., Ritrovato D., Reilly M.C., Naclerio M., Ranjan P., Kleebach J., Joshi P. (2021). Healthcare resource use and indirect costs associated with migraine in Italy: Results from the *My Migraine Voice* survey. J. Med. Econ..

[11] Ng Q.X., Zhou K.X., Lin C.Y.X., Chan C.K.M., Murugasu S., Ang A.Y.H., Sandhu G.S., Lim Y.L., Tang A.S.P. (2026). The Social Life of Migraine: A Meta-Ethnography of Lived Experience in Adults With Migraine. Health Serv. Insights.

[12] Rubin R. (2023). It Takes an Average of 17 Years for Evidence to Change Practice—The Burgeoning Field of Implementation Science Seeks to Speed Things Up. JAMA.

[13] Harris P.A., Taylor R., Minor B.L., Elliott V., Fernandez M., O’Neal L., McLeod L., Delacqua G., Delacqua F., Kirby J. (2019). The REDCap consortium: Building an international community of software platform partners. J. Biomed. Inform..

[14] Giannini G., Favoni V., Bauleo S., Ferrante T., Pierangeli G., Albani F., Reggiani M.L.B., Baruzzi A., Cortelli P., Cevoli S. (2012). SPARTACUS: Underdiagnosis of chronic daily headache in primary care. Neurol. Sci..

[15] Duburcq A., Molins M., Debremaeker S., Joie J., Lopes L., Nevoret C., de Dhaem O.B. (2025). Overview of migraine care, impact of the disease, and patient experience in France through patient voices: A cross-sectional and participatory survey study. J. Headache Pain.

[16] Vaghi G., De Icco R., Tassorelli C., Goadsby P.J., Vicente-Herrero T., de la Torre E.R. (2023). Who cares about migraine? Pathways and hurdles in the European region-access to care III. J. Headache Pain.

[17] Seng E., Lampl C., Viktrup L., Lenderking W.R., Karn H., Hoyt M., Kim G., Ruff D., Ossipov M.H., Vincent M. (2024). Patients’ Experiences During the Long Journey Before Initiating Migraine Prevention with a Calcitonin Gene-Related Peptide (CGRP) Monoclonal Antibody (mAb). Pain Ther..

[18] Ashraf U., Tomlinson S.E. (2025). Delays in migraine diagnosis and treatment: An Australian hospital headache clinic experience. Cephalalgia Rep..

[19] Peres M.F.P., Swerts D.B., de Oliveira A.B., Silva-Neto R.P. (2019). Migraine patients’ journey until a tertiary headache center: An observational study. J. Headache Pain.

[20] Becker W.J. (2017). The Diagnosis and Management of Chronic Migraine in Primary Care. Headache.

[21] Shapiro R.E., Muenzel E.J., Nicholson R.A., Zagar A.J., Reed M.L., Buse D.C., Hutchinson S., Ashina S., Pearlman E.M., Lipton R.B. (2025). Factors and Reasons Associated with Hesitating to Seek Care for Migraine: Results of the OVERCOME (US) Study. Neurol. Ther..

[22] Headache Classification Committee of the International Headache Society (IHS) (2018). The International Classification of Headache Disorders, 3rd edition. Cephalalgia.

[23] Eigenbrodt A.K., Ashina H., Khan S., Diener H.-C., Mitsikostas D.D., Sinclair A.J., Pozo-Rosich P., Martelletti P., Ducros A., Lantéri-Minet M. (2021). Diagnosis and management of migraine in ten steps. Nat. Rev. Neurol..

[24] Evans R.W., Burch R.C., Frishberg B.M., Marmura M.J., Mechtler L.L., Silberstein S.D., Turner D.P. (2019). Neuroimaging for Migraine: The American Headache Society Systematic Review and Evidence-Based Guideline. Headache.

[25] Perrone V., Leonetti S., Degli Esposti L., Giannessi L., Guerzoni S. (2026). Clinical and economic burden of migraine in Italy: A real-world evidence analysis. Glob. Reg. Health Technol. Assess..

